# Five new species of *Trigonopeltastes* Burmeister and Schaum from Central America with new country records for other New World Trichiini (Coleoptera, Scarabaeidae, Cetoniinae)

**DOI:** 10.3897/zookeys.617.9178

**Published:** 2016-09-15

**Authors:** Andrew B. T. Smith

**Affiliations:** 1 Research Division, Canadian Museum of Nature, P.O. Box 3443, Station D, Ottawa, Ontario, K1P 6P4, Canada

**Keywords:** Taxonomy, Central America, Mexico, Scarabaeoidea, Trichiina

## Abstract

Five new species of *Trigonopeltastes* Burmeister and Schaum, 1840 are described: *Trigonopeltastes arborfloricola* **sp. n.** from Nicaragua, *Trigonopeltastes formidulosus* **sp. n.** from Costa Rica, *Trigonopeltastes henryi* **sp. n.** from Costa Rica, *Trigonopeltastes mombachoensis* **sp. n.** from Nicaragua, and *Trigonopeltastes warneri* **sp. n.** from Belize and Guatemala. An updated key to species of *Trigonopeltastes* is presented. *Trigonopeltastes nigrinus* Bates, 1889 and *Trigonopeltastes carus* Bates, 1889 are placed in synonymy with *Trigonopeltastes geometricus* Schaum, 1841, **syn. n.**. The males of *Trigonopeltastes aurovelutinus* Curoe, 2011 and *Trigonopeltastes simplex* Bates, 1889 are described for the first time.

New country records are given for the following: *Giesbertiolus ornatus* Howden, 1988: Costa Rica; *Paragnorimus sambucus* Howden, 1970: Guatemala; *Trichiotinus bibens* (Fabricius, 1775): Canada; *Trigonopeltastes archimedes* Schaum, 1841: Guatemala and Costa Rica; *Trigonopeltastes frontalis* Bates, 1889: Belize, Guatemala, and Honduras; *Trigonopeltastes glabellus* Howden, 1988: Guatemala; *Trigonopeltastes geometricus* Schaum, 1841: Honduras; *Trigonopeltastes sallaei sallaei* Bates, 1889: Guatemala and Honduras; *Trigonopeltastes simplex* Bates, 1889: Mexico; *Trigonopeltastes variabilis* Howden, 1968: Honduras.

## Introduction

The genus *Trigonopeltastes* Burmeister and Schaum, 1840 (Coleoptera: Scarabaeidae: Cetoniinae: Trichiini: Trichiina) is distributed from the southern United States of America to northern Argentina, with most species occurring in Mexico and Central America. Specimens are most often collected in flowers of various shrubs and trees and also turn up in flight intercept traps and by beating vegetation. The rarity of many species in natural history collections emphasizes the need for targeted collecting efforts using specific methods in order to survey New World Trichiini fauna.

The taxonomy of the genus was modernized by Howden (1968), which allowed additional species to be discovered and described by Howden (1988), Howden and Ratcliffe (1990), Howden and Joly (1998), Ricchiardi (2003), and Curoe (2011). While curating and identifying specimens in natural history collections, I discovered five new species of *Trigonopeltastes* from Belize, Guatemala, Nicaragua, and Costa Rica. In addition, the examination of more specimens and longer series added several significant new distributional records and helped to clarify the taxonomic status of *Trigonopeltastes nigrinus* Bates, 1889.

The purpose of this paper is to describe five new species of *Trigonopeltastes*, update the identification key for *Trigonopeltastes*, synonymize *Trigonopeltastes nigrinus* with *Trigonopeltastes geometricus* Schaum, 1841, report new country records in the New World for a number of Trichiini species, and describe the previously unknown males of *Trigonopeltastes aurovelutinus* Curoe, 2011 and *Trigonopeltastes simplex* Bates, 1889. The genus *Trigonopeltastes* now contains 26 species.

## Materials and methods

### Specimens

More than 500 specimens were examined to form the basis of this review. The following institution and private collections (curators in brackets) are cited in the text as repositories for specimens. The term allotype follows the definition of Santiago-Blay et al. (2008).

CMNC
Canadian Museum of Nature, Ottawa, Ontario, Canada (François Génier, Robert Anderson) 


DCCC
 David C. Carlson Collection, Fair Oaks, California, United States of America 


DEBU
 Insect Collection, University of Guelph, Guelph, Ontario, Canada (Steve Paiero, Steve Marshall) 


EMEC
Essig Museum of Entomology, University of California, Berkeley, California, United States of America (Cheryl Barr) 


FSCA
 Florida State Collection of Arthropods, Gainesville, Florida, United States of America (Paul Skelley) 


MNCR
Museo Nacional de Costa Rica, San José, Costa Rica (formerly at Instituto Nacional de Biodiversidad (INBio), Santo Domingo de Heredia, Costa Rica) (Angel Solís) 


RACC
 Rich A. Cunningham Collection, Chino, California, United States of America 


SEMC
 Snow Entomological Museum, University of Kansas, Lawrence, Kansas, United States of America (Zack Falin) 


UNSM
University of Nebraska State Museum, Lincoln, Nebraska, United States of America (Brett Ratcliffe, M.J. Paulsen) 


USNM
 United States National Museum, Washington, District of Columbia, United States of America (currently housed at UNSM) (Brett Ratcliffe, M.J. Paulsen) 


### Label data, specimen images, and maps

The verbatim label data is given for specimens in quotation marks with slashes to indicate a new line of text on the label. The specimen images were taken at the CMNC using Leica imaging equipment and the Leica Application Suite software. The maps were created using the SimpleMappr website (http://www.simplemappr.net/). The specimen images and maps were modified and plates constructed in Adobe Photoshop.

## Taxonomy

### Trigonopeltastes arborfloricola sp. n.

Taxon classificationAnimaliaColeopteraScarabaeidae

http://zoobank.org/111C866F-E384-4251-BCF7-18D3B9EA932E

#### Type locality.

Reserva Privada Silvestre Domitila, Granada, Nicaragua.

#### Type series.

Holotype male at USNM labeled “NICARAGUA: Granada Prov. / Reserva Privada Silvestre / Domitila, 70 meters / 11°42.51'N, 85°57.16'W / 10 June 2007 N.E. Woodley / S.W. Lingafelter - tree flowers” (typeset). The holotype bears my red holotype label.

#### Description of holotype

**Figures 1–6.**
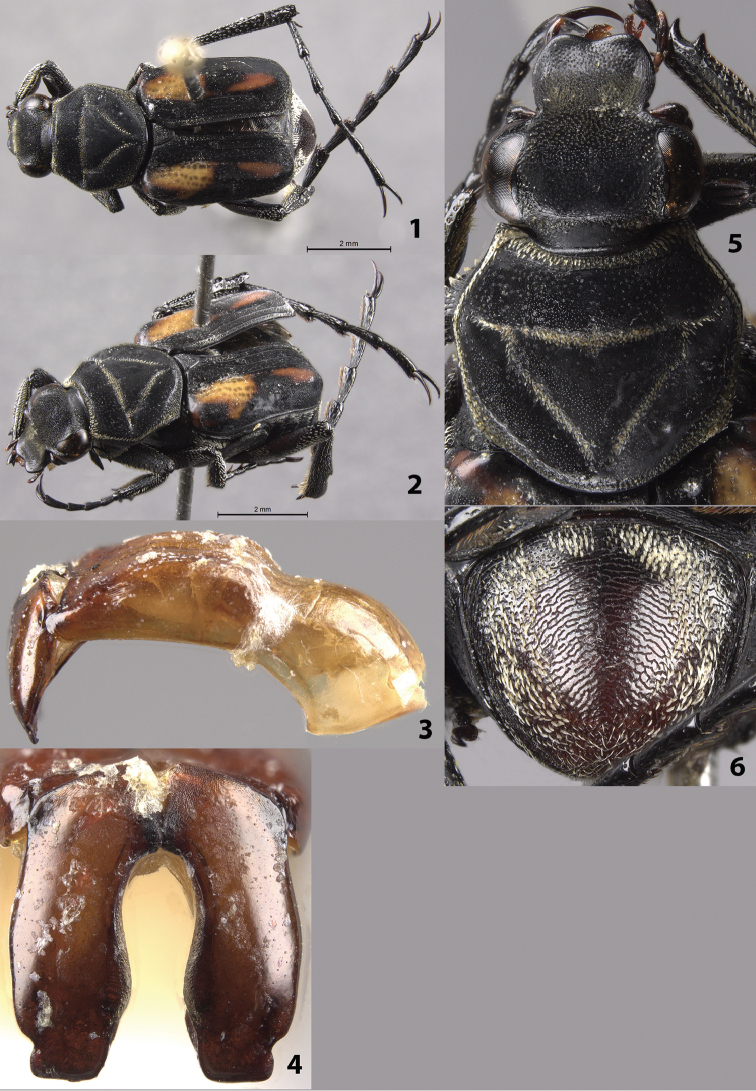
*Trigonopeltastes arborfloricola* sp. n., male holotype. **1** Dorsal habitus **2** Oblique habitus **3** Lateral genitalia **4** Parameres **5** Head and pronotum **6** Pygidium.

(Figs 1–6). Male. Length 9.0 mm, width 4.0 mm. Color: dorsal surface black with orange markings on elytra and light scales on pronotum, pygidium (Figs 1–2, 5–6). Mesofemur and metafemur tan along outside half, black along inside half; profemur, tarsi black. *Head*: Surface with short, dense setae medially on disc (sparsely setose to glabrous on apex of clypeus, base of frons) (Fig. 5), clypeus without longitudinally elongate punctures (punctures on head dense, round). Clypeus slightly wider than long with midline not elevated, apex emarginate. Antenna with 10 antennomeres, club length slightly shorter than length of antennomeres 2–7. Maxilla with long, thin brush protruding beyond clypeus in dorsal view. Mentum densely setose, obscuring surface. *Pronotum*: Surface of disc dull-black with shiny micropunctures. Marginal bead complete (obscured mediobasally), with complete ring of short, dense, yellow setae inside marginal bead. Pronotal disc with more-or-less complete inverted triangle grooved into the surface; groove with short, dense, yellow setae (Fig. 5). *Scutellum*: Surface with short, dense, white setae. *Elytra*: Surface glabrous, matt with shiny micropunctures, without cretaceous bands. Elytral striae 1–3 distinctly impressed, especially towards apex; remaining striae weakly defined with rows of punctures not impressed into surface. Orange markings consisting of a large mediobasal patch and smaller medioapical patch on each elytron (Figs 1–2). *Pygidium*: Surface without cretaceous makings, with complete ring of short, dense, yellow setae (Fig. 6). Disc with distinct, transverse microridges; surface evenly convex with apex slightly deflexed. *Venter*: Sternum and abdominal sternites without cretaceous markings, covered with dense, short, yellow setae. *Legs*: Protibia with 2 teeth near apex (Fig. 5). Mesotibia robust with edges weakly bowed outward medially. Tibial spurs acute, unmodified. Holotype with part of right protarsus missing. *Parameres*: Robust with lateral notches towards apex (Figs 3–4).

#### Etymology.

The name *arborfloricola* approximates “tree flower dweller” in Latin. The name is a noun in apposition. As indicated on the label, the holotype was collected in tree flowers.

#### Distribution

(Fig. 56). NICARAGUA - Granada (1): Reserva Privada Silvestre Domitila.

#### Temporal data.

June (1).

#### Remarks.

This species is fairly distinct in the complete lack of cretaceous markings and orange color pattern on the elytra. Hopefully more specimens will turn up in the lowlands of southern Nicaragua and northern Costa Rica so the intraspecific variation can be described.

### Trigonopeltastes formidulosus sp. n.

Taxon classificationAnimaliaColeopteraScarabaeidae

http://zoobank.org/7A845763-31AC-4A47-A969-C016851917C2

#### Type locality.

Monteverde, 1500 m, Puntarenas, Costa Rica.

#### Type series.

Holotype male, allotype female, 9 male paratypes, and 11 female paratypes. Holotype male, allotype female, and one male paratype at CMNC and one male paratype and one female paratype at UNSM labeled “COSTA RICA, Puntarenas / Monteverde, 1500m / May 11-13, 1996 / E. Giesbert, coll.” (typeset). One male and one female paratype at CMNC labeled “COSTA RICA, Puntarenas / San Luis (Monteverde) / 3900’ May 12-13, 1996 / E. Giesbert, coll.” (typeset). One male paratype (database # INBIOCRI000567265) at MNCR labeled “Est. G. Brenes, 1300m, / Res. Biol. Monteverde, / Prov. Punt. COSTA RICA / E. Bello, Jun 1991, / L-N-249750,450075” (typeset) and “Trigonopeltastes femoratus? / DET. / H. F. HOWDEN 94” (handwritten and typeset). One female paratype (database # INBIOCRI000601568) at MNCR labeled “Est. G. Brenes, 1300m, / Res. Biol. Monteverde, / Prov. Punt. COSTA RICA / E. Bello, Jun 1991, / L-N-249750,450075” (typeset) and “ADN Barcodeado / 2011 / Elena Ulate A.” (typeset). One male paratype (database # INBIOCRI001857744) at MNCR labeled “San Luis, Monteverde, R. B. Monteverde, / A. C. Arenal, Prov. Punta, COSTA RICA. / 1000-1350 m. Ene 1994, Z. Fuentes, L N / 449250_250850 # 2609” (typeset) One female paratype (database # INBIOCRI001894631) at MNCR labeled “Buen Amigo, San Luis Monteverde, A. C. / Arenal, Prov. Punta, COSTA RICA. 1000- / 1350 m. May 1994, Z. Fuentes, L N / 250850_449250 # 2926” (typeset). One male paratype (database # INBIOCRI001895044) at MNCR labeled “Buen Amigo, San Luis Monteverde, A. C. / Arenal, Prov. Punta, COSTA RICA. 1000- / 1350 m. May 1994, Z. Fuentes, L N / 250850_449250 # 2926” (typeset) and “ADN Barcodeado / 2011 / Elena Ulate A.” (typeset). One female paratype (database # INBIOCRI001895048) at MNCR labeled “Buen Amigo, San Luis Monteverde, A. C. / Arenal, Prov. Punta, COSTA RICA. 1000- / 1350 m. May 1994, Z. Fuentes, L N / 250850_449250 # 2926” (typeset) and “ADN Barcodeado / 2011 / Elena Ulate A.” (typeset). One male paratype (database # INBIOCRI001895061) at MNCR labeled “Buen Amigo, San Luis Monteverde, A. C. / Arenal, Prov. Punta, COSTA RICA. 1000- / 1350 m. May 1994, Z. Fuentes, L N / 250850_449250 # 2926” (typeset). One female paratype (database # INBIOCRI001923144) at MNCR labeled “San Luis, Monteverde, R. B. Monteverde / A. C. Arenal, Prov. Punta, COSTA RICA. / 1000-1350 m, 20-27 Jun 1994, Z. / Fuentes, L N 250850_449250 # 3029” (typeset). One female paratype (database # INBIOCRI001923145) at MNCR labeled “San Luis, Monteverde, R. B. Monteverde / A. C. Arenal, Prov. Punta, COSTA RICA. / 1000-1350 m, 20-27 Jun 1994, Z. / Fuentes, L N 250850_449250 # 3029” (typeset). One female paratype (database # INBIOCRI001923255) at MNCR labeled “Buen Amigo, San Luis Monteverde, A. C. / Arenal, Prov. Punta, COSTA RICA. 1000- / 1350 m. 8-12 Jun 1994, K. Matínez, L N / 250850_479250 # 3078” (typeset). One male paratype (database # INBIOCRI001992059) at MNCR labeled “Buen Amigo, San Luis Monteverde, Prov. / Punta, COSTA RICA. 1000 - 1350 m. Ago / 1994, Z. Fuentes, L N 250850_449250 # / 3168” (typeset), “Trigonopeltastes simplex Bates? / DET. / H. F. HOWDEN 01” (handwritten and typeset), and “ADN Barcodeado / 2011 / Elena Ulate A.” (typeset). One female paratype (database # INBIOCRI001992060) at MNCR labeled “Buen Amigo, San Luis Monteverde, Prov. / Punta, COSTA RICA. 1000 - 1350 m. Ago / 1994, Z. Fuentes, L N 250850_449250 # / 3168” (typeset) and “ADN Barcodeado / 2011 / Elena Ulate A.” (typeset). One female paratype (database # INBIOCRI000457825) at MNCR labeled “Fca. Cafrosa, 1300m. Est / Las Mellizas, P. Internac. / La Amistad, Prov. Punt. / COSTA RICA. M. / Ramirez, Jun 1991, / L-S-316100, 596100 ♀” (typeset and handwritten), “Trigonopeltastes / femoratus? / DET. / H.F. HOWDEN 94” (handwritten and typeset), and “ADN Barcodeado / 2011 / Elena Ulate A.” (typeset). One male paratype at UNSM labeled “COSTA RICA, PUNTARENAS / MONTEVERDE / APR 19-26 1988 / E. GIESBERT, COLL.” (handwritten) and “Tr. / nigrina / DET. group. / H.F. HOWDEN 91” (handwritten and typeset). One female paratype at UNSM labeled “COSTA RICA, PUNTARENAS / MONTEVERDE / APR 19-26 1988 / E. GIESBERT, COLL.” (handwritten). The types listed above bear my red holotype or allotype label or yellow paratype label.

#### Description of holotype

**Figures 7–13.**
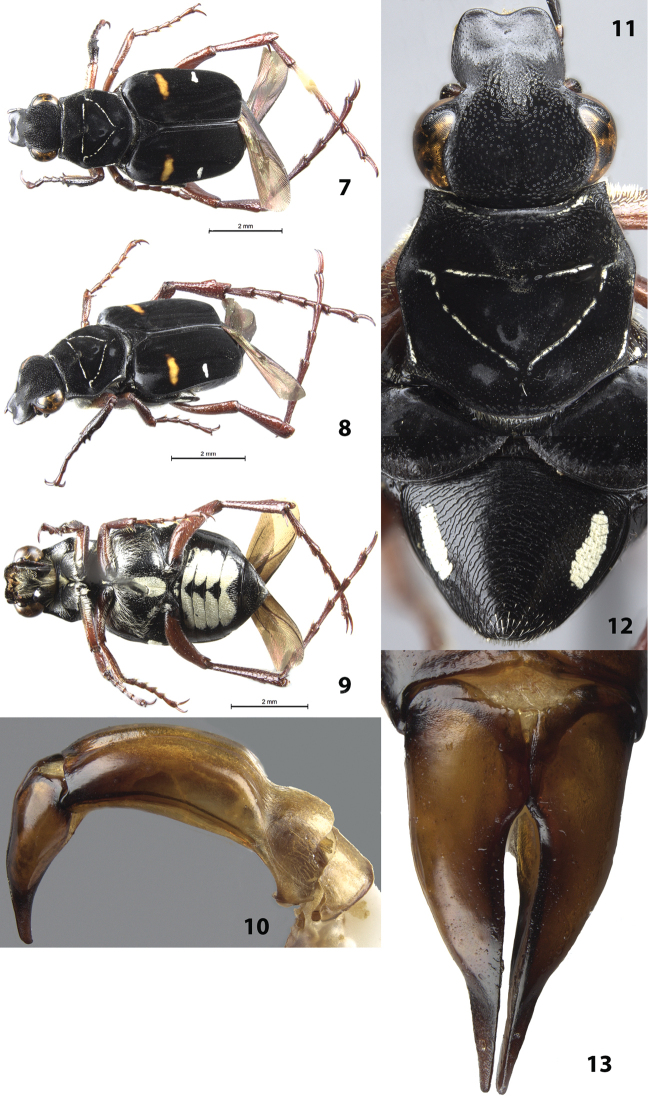
*Trigonopeltastes formidulosus* sp. n., male holotype. **7** Dorsal view of habitus **8** Oblique view of habitus **9** Ventral view of habitus **10** Lateral view of genitalia **11** Head and pronotum **12** Pygidium (male paratype used for this photograph) **13** Parameres.

(Figs 7–11, 13). Male. Length 9.0 mm, width 3.5 mm. Color: dorsal surface black with orange band and white, cretaceous band on each elytron (Figs 7–8). Legs tan except protibia and protarsus dark brown. *Head*: Surface setose with short setae medially on disc, without cretaceous markings (Fig. 11), clypeus with longitudinally elongate punctures. Clypeus about as long as wide with midline weakly elevated, apex emarginate. Antenna with 10 antennomeres, club longer than length of antennomeres 2–7. Maxilla with long, thin brush slightly protruding beyond clypeus in dorsal view. Mentum densely setose, obscuring surface. *Pronotum*: Surface of disc dull-black with shiny micropunctures (Fig. 11). Marginal bead complete, without complete ring of setose cretaceous markings inside marginal bead (only a few small patches of cretaceous markings in marginal bead). Pronotal disc with more-or-less complete inverted triangle indented into the surface with thin, cretaceous markings; cretaceous markings of triangle without setae (Fig. 11). *Scutellum*: Surface without cretaceous markings. *Elytra*: Surface glabrous, matt. Transverse cretaceous bands (1 on each side) adjacent to lateral edge approximately halfway between base and apex, length approximately ¼ width of single elytron (Figs 7–8). Elytral striae weakly defined with rows of small punctures, striae not indented into surface. Orange markings consisting of a transverse band adjacent to lateral edge approximately halfway between base and apex, width slightly less that half the width of single elytron (Figs 7–8). *Pygidium*: Surface with large, basiolateral, cretaceous makings; disc with distinct ridges of concentric circles (as in Fig. 12). Disc strongly, evenly convex with apex deflexed, flat. *Venter*: Sternum with large, central, cretaceous marking and smaller transverse, cretaceous markings apicolaterally and basiolaterally; remainder of surface setose (Fig. 9). Visible abdominal sternites 1–4 medially covered with cretaceous markings (except for small, central triangles); with short, white setae scattered throughout (Fig. 9). *Legs*: Protibia with 2 distinct teeth near apex (Fig. 8). Mesotibia robust with edges weakly bowed outward medially. Metatibia clavate. Tibial spurs acute, unmodified. *Parameres*: Robust at base tapering to an acuminate tip apically (Figs 10, 13).

#### Variation.

**Figures 14–17.**
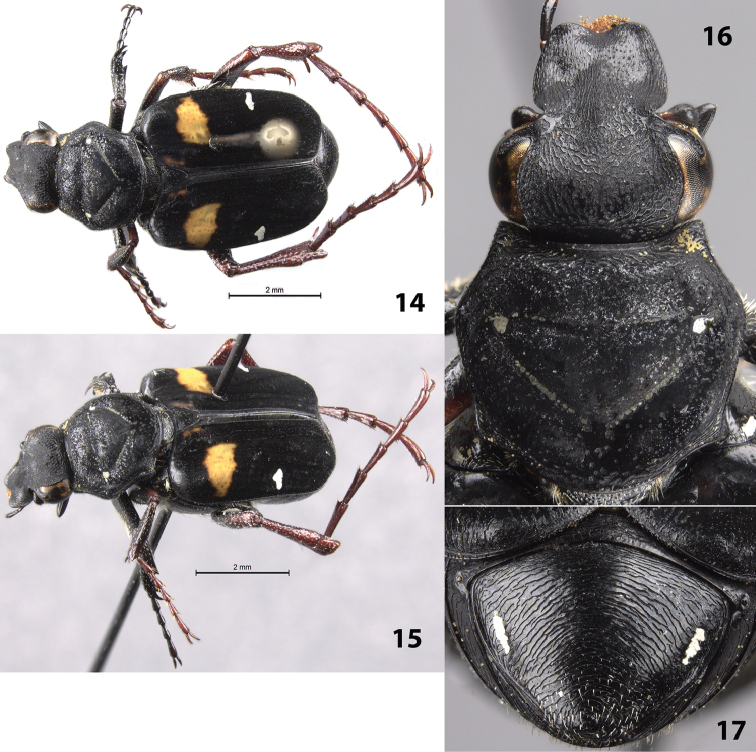
*Trigonopeltastes formidulosus* sp. n., female allotype. **14** Dorsal view of habitus **15** Oblique view of habitus **16** Head and pronotum **17** Pygidium.

Female allotype (Figs 14–17): length 9.5 mm, width 4.0 mm. The female allotype differs in the following characters. Color: orange band on elytra twice as thick as on holotype (Figs 14–15). Legs dark brown except mesotarsus, metatibia, and metatarsus tan. *Pygidium*: Surface with smaller basiolateral cretaceous makings. Disc weakly convex with apex not deflexed (Fig. 17). *Venter*: Sternum without cretaceous markings. Visible abdominal sternite 1 with small, lateral, cretaceous spot; sternites 2–4 without cretaceous markings. *Legs*: Protibia with 3 distinct teeth, 2 near apex and 1 medial. Paratypes: length 8.5–10.0 mm. Orange band on elytra either thick or thin, regardless of sex; 3 females had greatly expanded, orange coloration covering much of basal half of elytra. Legs vary from dark tan to dark brown with females generally having darker legs than males. *Pygidium*: Basiolateral cretaceous makings never significantly larger than seen in Figs 12, 17. *Venter*: Sternum and abdominal sternites of males with variable-sized cretaceous markings but markings always present in males and never present in females (except on sternite 1).

#### Etymology.

This species is named for the black-and-orange, Halloween-themed coloration of the dorsal surface. The word *formidulosus* is a Latin for “scary”. This name is an adjective in the nominative singular.

#### Distribution

(Fig. 56). COSTA RICA - Puntarenas (18): Buen Amigo (San Luis, Monteverde), Estación G. Brenes (Reserva Biológica Monteverde), Finca Cafrosa (Estación Las Mellizas, La Amistad), Monteverde, San Luis (Monteverde).

#### Temporal data.

January (1), May (9), June (6), August (2).

#### Remarks.

This species has many similarities to *Trigonopeltastes geometricus* but has consistently different dorsal color pattern and pygidial structure and cretaceous markings. *Trigonopeltastes geometricus* is variable across it’s distribution and within populations but typically has more extensive orange color patterns on the elytra and always has thick lateral bands of cretaceous markings on the pygidium often leaving only a medial strip exposed. *Trigonopeltastes formidulosus* consistently has a single orange spot on each elytron without further orange lines and markings (except for the occasional thin basal line adjacent to scutellum and three females have more extensive orange coloring covering much of the basal half of the elytra). The apex of the pygidium in males is deflexed at a 90° angle in *Trigonopeltastes formidulosus* while only slightly convex in male *Trigonopeltastes geometricus*.

### Trigonopeltastes henryi sp. n.

Taxon classificationAnimaliaColeopteraScarabaeidae

http://zoobank.org/2E3117D5-BB7E-4986-A520-D3F4E42735BC

#### Type locality.

San Luis (south of Monteverde), Puntarenas, Costa Rica.

#### Type series.

Holotype male and allotype female. Holotype male at UNSM labeled a) “COSTA RICA, Puntarenas / San Luis (Monteverde) / 3900’ May 12-13, 1996 / E. Giesbert, coll.” (typeset). Allotype female at CMNC labeled a) “Prov. SAN JOSE / San Ant. / Desamp. 1 Mayo 1976 / Col. U. Ureña.” (typeset and handwritten), b) “H. & A. HOWDEN / COLLECTION / Ottawa, Canada” (typeset). Both types bear my red holotype or allotype label.

#### Description of holotype

**Figures 18–23.**
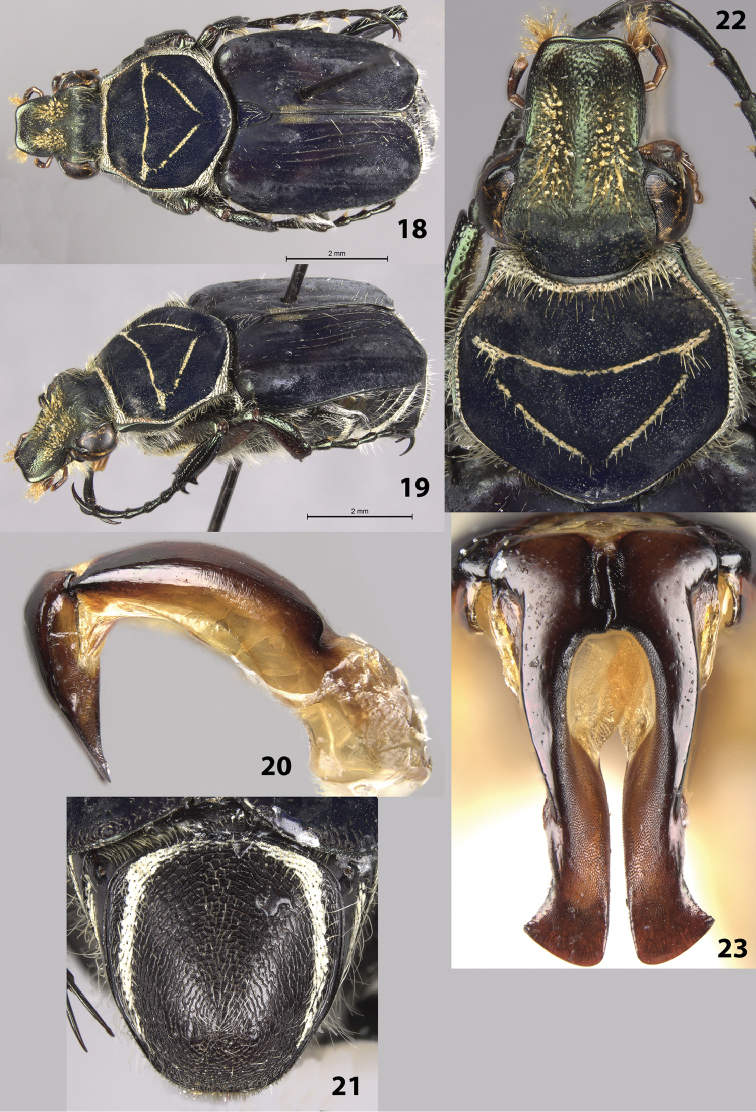
*Trigonopeltastes henryi* sp. n., male holotype. **18** Dorsal view of habitus **19** Oblique view of habitus **20** Lateral view of genitalia **21** Pygidium **22** Head and pronotum **23** Parameres.

(Figs 18–23). Male. Length 12.5 mm, width 4.5 mm. Color: head and legs shiny dark metallic green; pronotum and elytra with dull, dark blue, velvety appearance (Figs 18–19, 22); ventral surface shiny black. *Head*: Surface densely setose medially on disc (except along midline); apex, base, and midline of head moderately to sparsely setose (Fig. 22); clypeus with some longitudinally elongate punctures. Clypeus slightly longer than wide with margins and midline distinctly elevated, apex weakly emarginate. Head without cretaceous markings (Fig. 22). Antenna with 10 antennomeres, club length approximately equal to length of antennomeres 2–7. Maxilla with long, thin brush protruding beyond clypeus in dorsal view. Mentum densely setose, obscuring surface. *Pronotum*: Surface of disc dull-blue matt with shiny micropunctures (Fig. 22). Marginal bead complete, with complete ring of setose cretaceous markings inside marginal bead. Pronotal disc with more-or-less complete inverted triangle indented into the surface with cretaceous markings, diagonal lines of triangle with row of setae (Fig. 22). *Scutellum*: Surface setose, without cretaceous markings. *Elytra*: Surface sparsely setose, matt. Transverse cretaceous band (1 on each side) short, located in basal half of elytral suture (Figs 18–19, near pin but obscured by grease); weak cretaceous markings at apex of suture adjacent to pygidium. Elytral striae well defined with weak indentations between humeral angle and elytral suture. *Pygidium*: Surface rugose in a circular, finger-print pattern; densely setose (especially around margins and midline); with thin, inverted U-shaped cretaceous band along basal and lateral surfaces of disc (Fig. 21). Disc strongly, evenly convex. *Venter*: Sternum with numerous cretaceous markings, densely setose with long setae somewhat obscuring surface. Visible abdominal sternites 2–5 with transverse cretaceous bands thick medially, thin laterally; cretaceous bands setose. *Legs*: Protibia with 2 teeth near apex (Fig. 19). Mesotibia robust with outer edge bowed outward medially. Tibial spurs acute, unmodified. Tarsi with apicoventral tufts of setae. Holotype with metatarsal legs missing except for 1 metafemur. *Parameres*: Apically enlarged with a triangular lateral projection (Figs 20, 23).

#### Variation.

**Figures 24–27.**
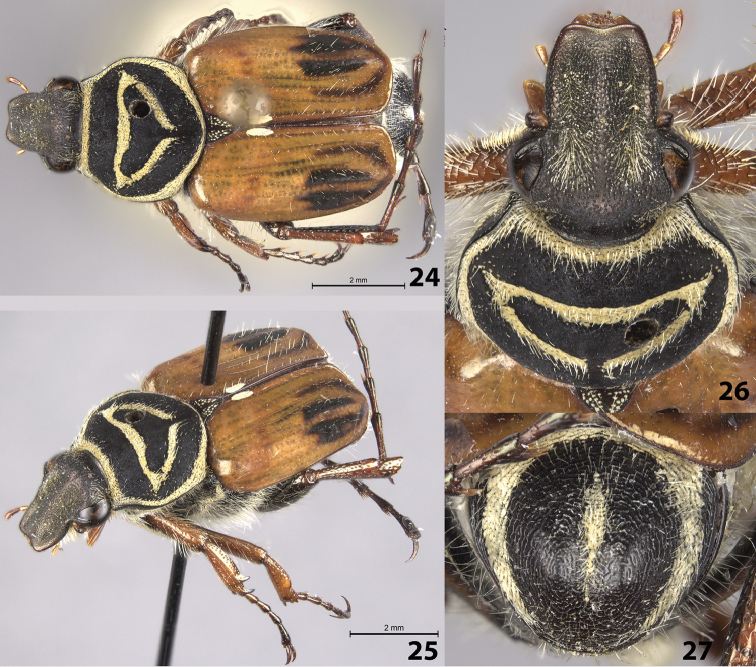
*Trigonopeltastes henryi* sp. n., female allotype. **24** Dorsal view of habitus **25** Oblique view of habitus **26** Head and pronotum **27** Pygidium.

Female allotype (Figs 24–27): length 12.0 mm, width 5.0 mm. The female allotype differs in the following characters. Color: dorsal surface of head bronze with weak green reflections; pronotum and scutellum matt black; elytra dull orange with dull black pattern on apical half (Figs 24–26). Legs tan, metafemur and parts of metatibia shiny black. *Pronotum*: Marginal bead and impressed triangle on disc with thicker cretaceous markings (Fig. 26). *Scutellum*: Surface with small cretaceous spots. *Pygidium*: Disc with longitudinally concave midline, cretaceous markings along midline (Fig. 27). *Venter*: Visible abdominal sternites 2–5 with transverse cretaceous bands thinner, of even thickness, interrupted medially.

#### Etymology.

This species is named for the late Henry Howden (Ottawa, Canada) as thanks for bringing the female allotype to my attention and to honor his great contributions to our knowledge of *Trigonopeltastes* and other Trichiini.

#### Distribution

(Fig. 56). COSTA RICA (2) - Puntarenas (1): San Luis (Monteverde); San José (1): San Antonio de Desamparados.

#### Temporal data.

May (2).

#### Remarks.

The male holotype and female allotype are the only known specimens of this new species. The very different dorsal coloration for these specimens is unusual but not unheard of within this genus. I do have some misgivings about placing these two specimens together as one species but decided to take this conservative approach because the structural characters are similar, the size and shape of the two specimens match well, the general cretaceous patterns are congruent, and the two specimens were collected within close proximity to one another. More specimens will need to be examined to understand the color variation within this species and to test my hypothesis that these male and female specimens belong to the same species.

### Trigonopeltastes mombachoensis sp. n.

Taxon classificationAnimaliaColeopteraScarabaeidae

http://zoobank.org/EA928CEE-BC17-4504-8584-6930FCB94F32

#### Type locality.

Reserva Nacional Volcán Mombacho, Granada, Nicaragua.

#### Type series.

Holotype male, allotype female, and 2 female paratypes. Holotype male at SEMC labeled a) “NICARAGUA: Granada Dept. / Res. Nat. Volcan Mombacho / 1150m 11°50.05'N 85°58.83'W / 3-VI-2002, R. Brooks, Z. Falin, / S. Chatzimanolis ex. misc. / collecting, NIC1BFC02 165” (typeset), b) “SMO533385 / KUNHM-ENT” (typeset beneath barcode). Allotype female at CMNC labeled “NICARAGUA: Grenada Dept. / Volcan Mombacho Res. Nat. / N11°50.0’ W85°58.8’, 1150 m / elfin cloud forest, beating, / 2-5.VI.2002, R.Anderson / RSA2002-034” (typeset). One female paratype at CMNC labeled “NICARAGUA: Grenada / Dept. Volcan Mombacho Res. / Nat. 1150 m, 11°50.0'N / 85°58.8'W 2-5.VI.2002, R. / Anderson, ex. elfin cloud forest / beating. RSA2002-034X” (typeset). One female paratype at CMNC labeled “NICARAGUA: Granada / Volcan Mombacho / Bosque nuboso #1 / 30-V-1998, malaise trap / J.M. Maes” (typeset). The types listed above bear my red holotype or allotype label or yellow paratype label.

#### Description of holotype

**Figures 28–33.**
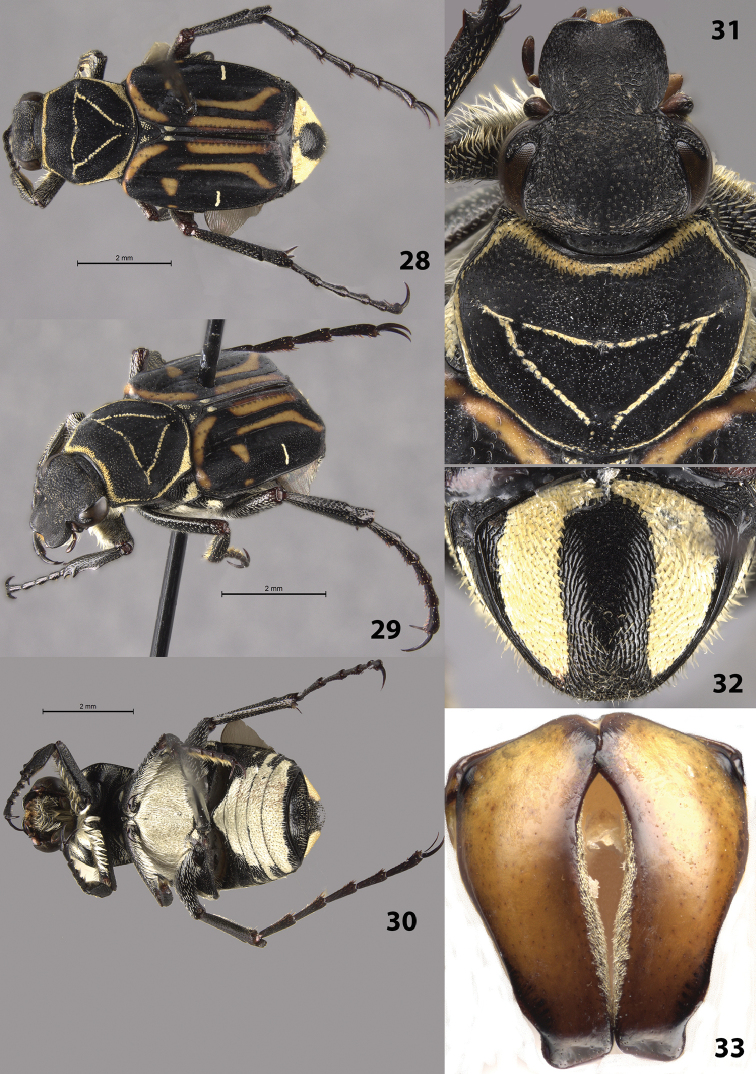
*Trigonopeltastes mombachoensis* sp. n., male holotype. **28** Dorsal view of habitus **29** Oblique view of habitus **30** Ventral view of habitus **31** Head and pronotum **32** Pygidium **33** Parameres.

(Figs 28–33). Male. Length 9.0 mm, width 4.0 mm. Color: dorsal surface black with orange markings on elytra and light scales on pronotum, elytra, pygidium (Figs 28–29). Legs black. *Head*: Surface glabrous, clypeus without longitudinally elongate punctures (punctures on head dense, round) (Fig. 31). Clypeus slightly wider than long with midline not elevated, apex emarginate. Antenna with 10 antennomeres, club length approximately equal to length of antennomeres 2–7. Maxilla with long, thin brush protruding beyond clypeus in dorsal view. Mentum densely setose, obscuring surface. *Pronotum*: Surface of disc dull-black with shiny micropunctures (Fig. 31). Marginal bead complete (obscured mediobasally); with complete ring of short, dense, yellow setae and cretaceous markings inside marginal bead. Pronotal disc with more-or-less complete inverted triangle indented into surface; indentation with light cretaceous markings (Fig. 31). *Scutellum*: Surface with oblique, cretaceous markings on each side. *Elytra*: Surface glabrous, matt with shiny micropunctures. Transverse cretaceous band (1 on each side) short, located on basal half of elytral suture. Cretaceous bands (1 on each side) adjacent to lateral edge approximately halfway between base and apex, length approximately ¼ width of elytron (Figs 28–29). Elytral striae 1–4 weakly impressed; remaining striae weakly defined with rows of punctures, not impressed into surface. Orange markings consisting of transverse, basal band; sub-basal, medial spot; 2 longitudinal, medioapical lines (Figs 28–29). *Pygidium*: Surface with thick, lateral bands of cretaceous makings that are narrowly joined basally; non-cretaceous area of disc setose, especially near apex (Fig. 32). Disc with distinct, concentric microridges; surface evenly convex with apex slightly deflexed. *Venter*: Metasternum entirely covered with cretaceous markings and light setae, abdominal sternites with broad, medial cretaceous markings and light setae (Fig. 30). *Legs*: Protibia with 2 teeth near apex, 1 broad medial tooth (Fig. 29). Mesotibia robust with edges weakly bowed outward medially. Tibial spurs acute, unmodified. Holotype with right mesotarsomeres 2–5 missing. *Parameres*: Robust with weak lateral notches towards apex (Fig. 33).

#### Variation.

**Figures 34–37.**
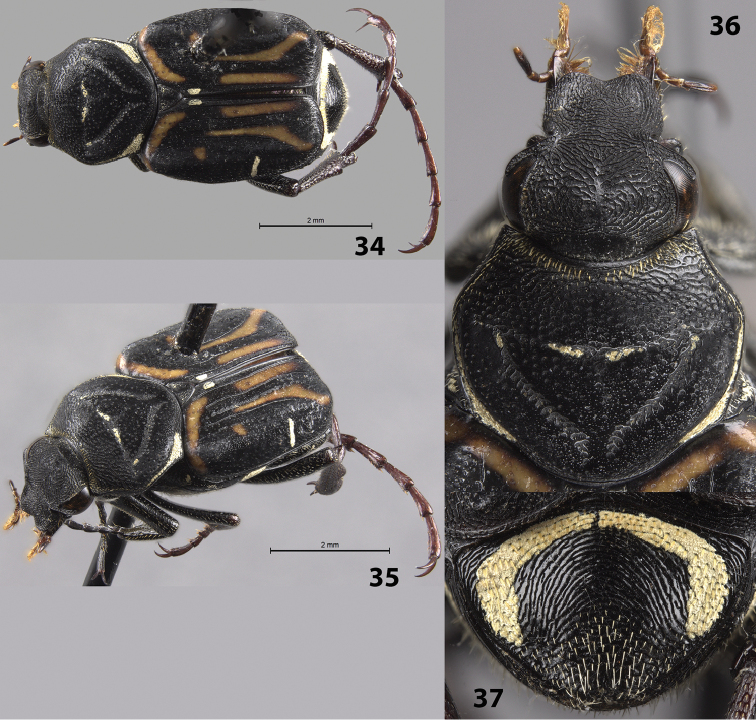
*Trigonopeltastes mombachoensis* sp. n., female allotype. **34** Dorsal view of habitus **35** Oblique view of habitus **36** Head and pronotum **37** Pygidium.

Female allotype (Figs 34–37): length 8.5 mm, width 3.0 mm. The female allotype differs in the following characters: *Pronotum*: Pronotal indented triangle with minimal cretaceous markings (perhaps due to abrasion, 2 female paratypes have more prominent cretaceous markings in pronotal triangle) (Figs 34–36). *Pygidium*: Surface with much thinner, rounder lateral bands of cretaceous makings along basal and lateral margins (Fig. 37). Disc flat with apex not deflexed. *Venter*: Metasternum with greatly reduced, lateral cretaceous markings, abdominal sternites with reduced, lateral cretaceous markings. *Legs*: Protibia with 3^rd^ medial tooth larger and more distinct. Paratypes: length 9.0–10.0 mm. All characters are similar to those in the female allotype.

#### Etymology.

This species is named for Volcán Mombacho, where all known specimens were collected.

#### Distribution

(Fig. 56). NICARAGUA - Granada (4): Reserva Nacional Volcán Mombacho.

#### Temporal data.

May (1), June (3).

#### Remarks.

This species is similar to *Trigonopeltastes intermedius* but can be distinguished by the orange color pattern on the elytra and geographic distribution. *Trigonopeltastes intermedius* has a solid, transverse, sub-basal, orange line while *Trigonopeltastes mombachoensis* has a sub-basal, orange spot (Figs 28, 34). *Trigonopeltastes intermedius* is known from Mexico and Guatemala and *Trigonopeltastes mombachoensis* is only known from Volcán Mombacho, Nicaragua. Any specimens found of either species in Honduras or northern Nicaragua should be studied to see if these character states are transitional.

### Trigonopeltastes warneri sp. n.

Taxon classificationAnimaliaColeopteraScarabaeidae

http://zoobank.org/EF644BD9-86EB-44FB-B09C-DC1B9373D89F

#### Type locality.

Las Cuevas Research Station, Chiquibul National Forest, Cayo District, Belize.

#### Type series.

Holotype male, allotype female, and 1 male paratype. Holotype male and allotype female at FSCA labeled “BELIZE: Cayo District / Chiquibul N. F. / Las Cuevas Research Sta. / 16° 43’59"N, 88°59’11"W / 29.V.2003; J.A. Shuey” (typeset). One male paratype at CMNC labeled “GUATEMALA: Petén / Cerro Cahuí / 16.99876 -89.71038 ±206m / 150m, 24.V.2009 / LLAMA #Go-B-05-3-01 / tropical moist forest, beating veg” (typeset). The types listed above bear my red holotype or allotype label or yellow paratype label.

#### Description of holotype

**Figures 38–42.**
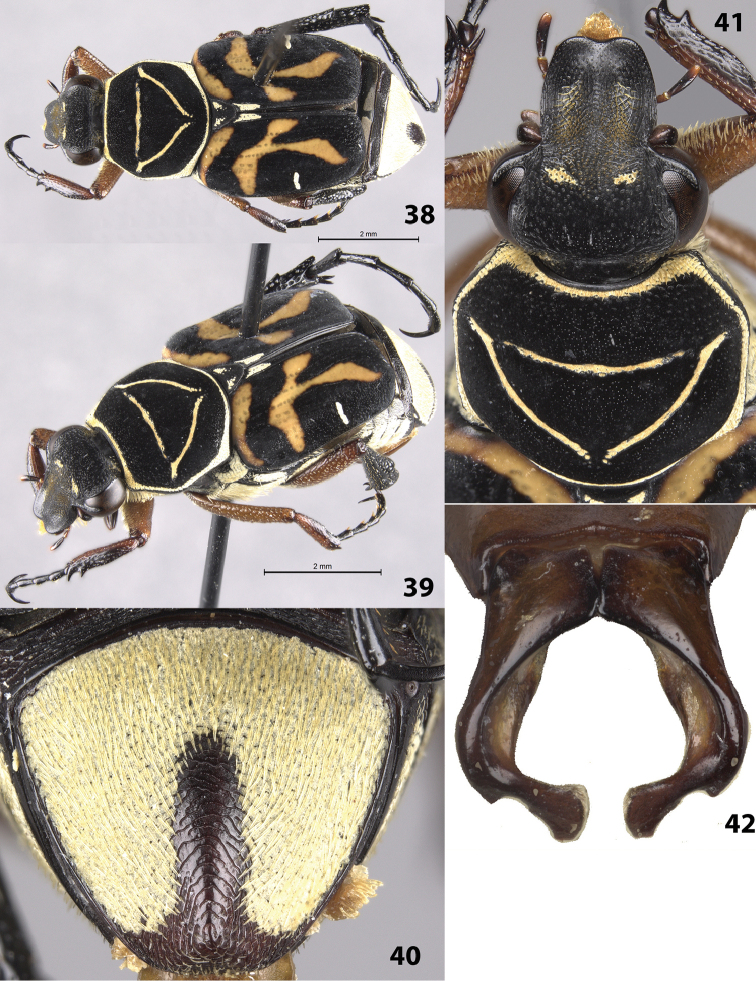
*Trigonopeltastes warneri* sp. n., male holotype. **38** Dorsal view of habitus **39** Oblique view of habitus **40** Pygidium **41** Head and pronotum **42** Parameres.

(Figs 38–42). Male. Length 9.5 mm, width 3.5 mm. Color: dorsal surface black with orange markings on elytra and yellow cretaceous markings on head, pronotum, scutellum, elytra, and pygidium (Figs 38–39, 40). Femora tan, protibia and mesotibia half tan and half black longitudinally, metatibia and tarsi black. *Head*: Surface densely setose with short setae medially on disc (except along midline), frons with paired cretaceous markings between eyes (Fig. 41), clypeus with longitudinally elongate punctures. Clypeus about as long as wide with midline distinctly elevated, apex emarginate. Antenna with 10 antennomeres, club length approximately equal to length of antennomeres 2–7. Maxilla with long, thin brush protruding beyond clypeus in dorsal view. Mentum densely setose, setae obscuring surface. *Pronotum*: Surface of disc dull-black with shiny micropunctures (Fig. 41). Marginal bead complete; with complete ring of setose, cretaceous markings inside marginal bead. Pronotal disc with more-or-less complete inverted triangle indented into the surface with cretaceous markings, cretaceous markings of triangle without setae (Fig. 41). *Scutellum*: Surface with cretaceous patches in basal corners. *Elytra*: Surface glabrous, matt. Transverse cretaceous band (1 on each side) short, located on basal half of elytral suture. Cretaceous bands (1 on each side) adjacent to lateral edge approximately halfway between base and apex, length approximately ¼ width of elytron (Figs 38–39). Elytral striae weakly defined with rows of punctures not indented into surface. Orange markings consisting of transverse, basal band and continuous, oblique T-shaped pattern on each elytron (Figs 38–39). *Pygidium*: Surface mostly covered with cretaceous makings except for apex and apical 2/3 of medial line (Fig. 40). Disc strongly, evenly convex with apex deflexed, flat. *Venter*: Sternum mostly covered with cretaceous markings and setae. Visible abdominal sternites 1–5 almost entirely covered with cretaceous markings, with erect setae scattered throughout. *Legs*: Protibia with 2 teeth near apex (Fig. 39). Mesotibia robust with outer edge weakly bowed outward medially. Tibial spurs acute, unmodified. Holotype with 1 protarsus and 1 metatarsus missing. *Parameres*: Strongly curved, roughly forming a circle; apex strongly curved inward, enlarged, dorsoventrally flattened (Fig. 42).

#### Variation.

**Figures 43–45.**
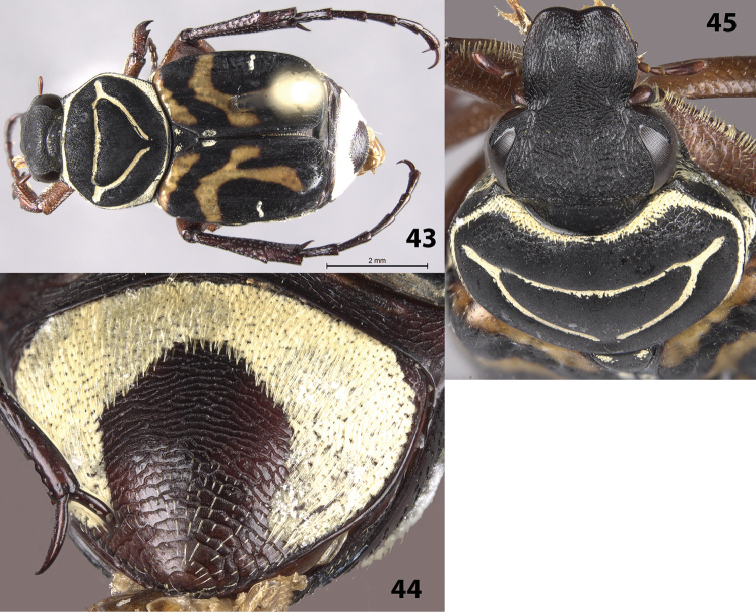
*Trigonopeltastes warneri* sp. n., female allotype. **43** Dorsal view of habitus **44** Pygidium **45** Head and pronotum.

Female allotype (Figs 43–45): length 9.0 mm, width 4.0 mm. The female allotype differs in the following characters: Color: Legs tan to brown. *Head*: Surface moderately setose with short, obscure setae, head without cretaceous markings (Fig. 45). *Pygidium*: Surface with thick, inverted U-shaped, cretaceous markings along lateral margins and base; apically and medially without cretaceous markings (Fig. 44). Disc evenly convex without deflexed apex. *Venter*: Sternum only partially covered with cretaceous markings. Visible abdominal sternites 1–5 without cretaceous markings medially. *Legs*: Protibia with 3 teeth, 2 near apex and 1 medial. Mesotibia without outer edge bowed outward. Male paratype: length 9.0 mm. Femora and mesotibia orange, protibia half orange and half black longitudinally, metatibia and tarsi black with some dark red. *Head*: Frons with single cretaceous marking extending between eyes. Clypeus with midline setose, not distinctly elevated. The male paratype is similar in all characters to the holotype.

#### Etymology.

This species is named after Bill Warner (Chandler, Arizona) as thanks for bringing the holotype and allotype specimens to my attention.

#### Distribution

(Fig. 57). BELIZE - Cayo (2): Las Cuevas Research Station, Chiquibul National Forest. GUATEMALA – Petén (1): Cerro Cahuí (16.99876°N, 89.71038°W).

#### Temporal data.

May (3).

#### Remark.

This species is similar externally to *Trigonopeltastes sallaei sallaei* and *Trigonopeltastes intermedius* but has differences in the elytral coloration pattern. *Trigonopeltastes warneri* has distinct male parameres (Fig. 42) and is found in the lowlands of eastern Guatemala and Belize, while *Trigonopeltastes sallaei sallaei* and *Trigonopeltastes intermedius* are typically found at mid to high elevation localities.

### Notable new distributional records, male descriptions, and new synonymies for New World Trichiini

#### Giesbertiolus ornatus

Taxon classificationAnimaliaColeopteraScarabaeidae

Howden, 1988

##### Distribution.

This species was previously recorded from Panama (Howden 1988). The specimens detailed below represent a new country record for Costa Rica.

“Buen Amigo, San Luis Monteverde, A. C. / Arenal, Prov. Punta, COSTA RICA, 1000- / 1350 m. May 1994, Z. Fuentes, L N / 250850_449250 # 2926” (1 male - MNCR).

“COSTA RICA, Prov. Alajuela, San / Cristobal. 600-620m. 18 MAY 1998. / F. A. Quesada. En Flores. / L_N_318056_383200 #50698” (1 male, 1 female - MNCR).

“COSTA RICA. Prov. Guanacaste, / Rincón de la Vieja, Upala, Dos Ríos / San Cristobal, 600-620m, 17 MAY / 1998, F. A. Quesada, En Flor. / I_N_318056_383200 #63528” (1 female - MNCR).

“Est. Cacao, 1000-1400m, / Lado SO Vol. Cacao, / P. N. G., Prov. Guan. / COSTA RICA, C. / Chaves, Jun 1991. / L-N-323300,375700” (1 female - MNCR).

“Est. Cacao, 1000-1400m, / Lado SO Vol. Cacao, P. N. / Guan., Prov. Guanacaste, / Costa Rica, Z. Fuentes, / 21 a 29 may 1992 / L-N 323300,375700” (1 female - MNCR).

“COSTA RICA, Puntarenas, Fca. / Buen Amigo Monteverde, 4Km S. / de la Reserva 1000-1350m. MAY / 1997, Z. Fuentes, Red Mariposa. / L_N_250850_449250 #46800” (1 female - MNCR).

“COSTA RICA, Prov. Alajuela, San / Cristobal. 600-620m. 18 MAY 1998. / F. A. Quesada. En Flores / L_N_318056_383200 #50698” (1 male - CMNC)

“COSTA RICA. Prov. Guanacaste / Rincón de la Vieja, Upala, Dos Ríos, / San Cristobal, 600 – 620m, 17 MAY / 1998. F. A. Quesada, En Flor, / L_N_318056_383200 #63528” (1 female - CMNC)

#### Paragnorimus sambucus

Taxon classificationAnimaliaColeopteraScarabaeidae

Howden, 1970

##### Distribution.

This species was previously recorded from Mexico (Howden 1970). The specimens detailed below represent a new country record for Guatemala.

“GUATEMALA, Huehuetenango / Nentón, Gracias a Dios, El / Quetzal 1600 m. 20-vi-2006 / J. Monzón y F. Camposeco / COLECCION J. MONZON” (1 male - CMNC).

“GUATEMALA. Huehuetenan- / go. Aguacatán. Río Sn. Juan / 2,212m 6 JUNIO 2009 / 15.368600 – 91.288930 / Col. José Monzón Sierra” (1 male - CMNC).

#### Trichiotinus bibens

Taxon classificationAnimaliaColeopteraScarabaeidae

(Fabricius, 1775)

##### Distribution.

This species was previously recorded from the United States of America (Howden 1968). The specimens detailed below represent a new country record for Canada. These specimens were likely collected about 100–150 years ago. More collecting is needed around London, Ontario and southwestern Ontario to further verify this record. William Saunders (1835–1914) lived in London from 1847–1886 (Bethune 1914) and was a founding member of the Entomological Society of Canada in 1863 and frequent contributor to the early issues of the *Canadian Entomologist*.

“London / W. Saunders” Canadian scarab database numbers CSD013086–CSD013089 (3 males, 1 female – DEBU)

#### Trigonopeltastes archimedes

Taxon classificationAnimaliaColeopteraScarabaeidae

Schaum, 1841

##### Distribution.

This species was previously recorded from Mexico and El Salvador (Howden 1968). The specimens detailed below represent new country records for Guatemala and Costa Rica.

“GUAT.: BAJA VERAPAZ / 54.4km S. Purulha, / 850m, 1.VII.1993, / F. Génier, hand coll.” (1 male - CMNC).

“GUATML. Zacapa 12-14 / km S Sn Lorenzo 1-2000’ / June 3-6 1989 / J. E. Wappes” (2 males - CMNC).

“GUAT., Zacapa Sn / Lorenzo Rd 1500- / 1800'1-10 June / 1991 JE Wappes” (1 male - CMNC).

“GUAT. Zacapa / 12km S. San Lorenzo / 510m 16.VI.1993 / H. & A. Howden” (1 male, 1 female - CMNC).

“COSTA RICA: Guanacaste / Prov., Comelco (50m) / 8 km NW Bagaces / VI-5-1973, P.A. Opler / on: Croton sp.” (1 male - CMNC).

“COSTA RICA: Guanacaste, / Parque Nacional Santa Rosa, / Estación Santa Rosa, 295 m. / N10°50'21.4", W 35°37'05.8" / 16-VII-2004 / Barney D. Streit, collector” (2 males, 1 female – CMNC, RACC)

“COSTA RICA: Guanacaste, / Parque Nacional Santa Rosa, / Estación Santa Rosa, / 17-VII-2004 / Barney D. Streit, collector” (2 females – CMNC, RACC)

“COSTA RICA: Guanacaste, / Parque Nacional Santa Rosa, / Estación Santa Rosa, 295 m. / N 10°50'21.4", W 35°37'05.8" / 4-VII-2005 / Barney D. Streit, collector” (2 males – CMNC, RACC)

#### Trigonopeltastes aurovelutinus

Taxon classificationAnimaliaColeopteraScarabaeidae

Curoe, 2011

##### Remarks.

The male of this species was previously unknown so a description of the key characters is given below. One male specimen was examined labeled “Mex: Guerrero / Acuhuezotla / IX-29-94 Chemsak” (EMEC).

##### Description of male

**Figures 46–51.**
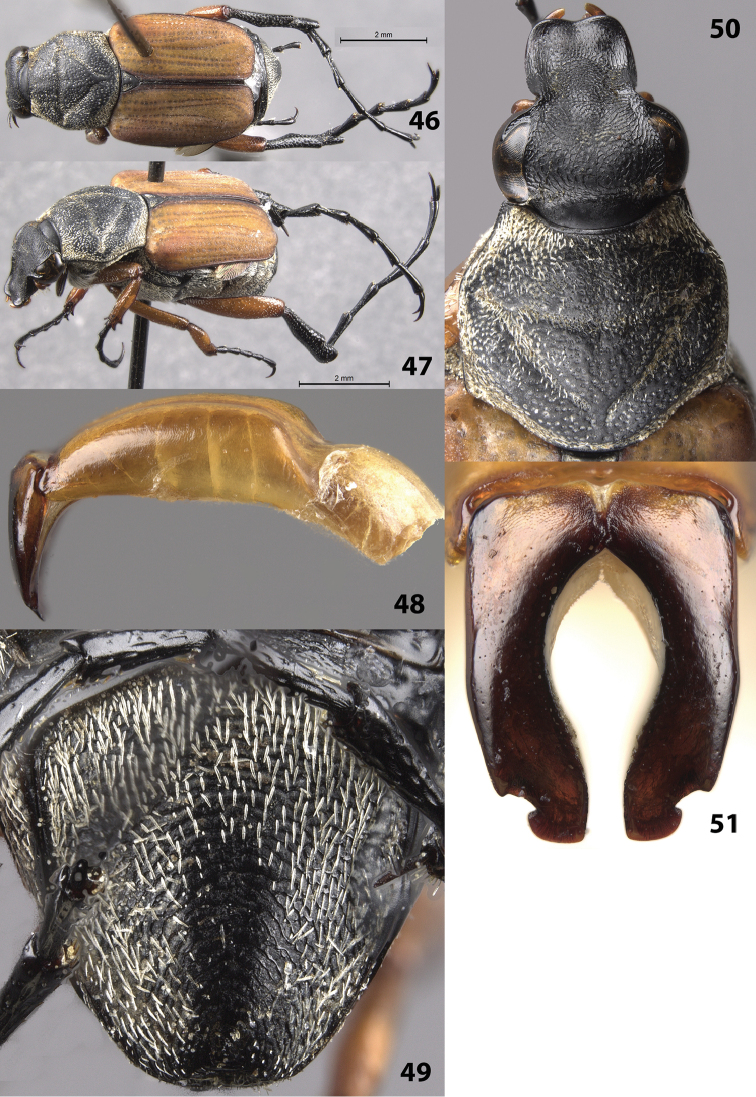
*Trigonopeltastes aurovelutinus* Curoe, 2011, male from EMEC (label data: “MEX: Guerrero / Acahuezotla / IX-29-94 Chemsak”) **46** Dorsal view of habitus **47** Oblique view of habitus **48** Lateral view of genitalia **49** Pygidium **50** Head and pronotum **51** Parameres.

(Figs 46–51). Color: similar to the description of the females (Curoe 2011; Figs 46–47); dorsal surface black except elytra uniformly orange with darker sutural line. Legs orange with black tarsi and metatibia (Fig. 47). *Head*: Surface glabrous except for light, setose patches at base of clypeus and apex of frons, without cretaceous markings (Fig. 50). *Pronotum*: Surface of disc dull-black with shiny micropunctures (Fig. 50); setae scattered across disc but not obscuring surface as seen in the Curoe (2011) figure of the female (perhaps due to abrasion). Marginal bead complete; with thick, scale-like setae. Pronotal disc with more-or-less complete inverted triangle indented into the surface with thick scale-like setae (Fig. 50). *Elytra*: Orange, glabrous, without cretaceous markings (Figs 46–47). *Pygidium*: Surface with white, scale-like setae covering disc (but not obscuring surface, perhaps due to abrasion), without cretaceous makings (Fig. 49). *Venter*: Surface covered with white, scale-like setae. Visible abdominal sternites with erect, white setae scattered medially. *Legs*: Protibia with 2 teeth near apex (Fig. 47) (female with third medial tooth; Curoe 2011). Metatibia with a distinct, medial protuberance along inner surface (Fig. 46), surface distad to this protuberance smooth with fine striations that may be for stridulation (a key diagnostic character not found in male *Trigonopeltastes simplex* and not apparent in the description and figure of the female *Trigonopeltastes aurovelutinus*). *Parameres*: Robust with lateral notches towards apex (Figs 48, 51).

#### Trigonopeltastes frontalis

Taxon classificationAnimaliaColeopteraScarabaeidae

Bates, 1889

##### Distribution.

This species was previously recorded from Mexico (Howden 1968) and El Salvador (Cave 1983). The specimens detailed below represent new country records for Belize, Guatemala, and Honduras.

“Augustine / Br. Honduras / July 3 1969 / F. D. Bennett” (1 male - CMNC). Augustine is in the Cayo District of Belize.

“GUAT., BAJA VERAPAZ / 5 KM S SAN JERONIMO / 4500’, MAY 24-30, 1989 / E. GIESBERT, COLL.” (1 male - CMNC)

“HONDURAS Olancho / Dept., P.N. La Muralla / ± 1200m, flowers / 1 July 1995 / DC Carlson/FT Hovore” (1 male - DCCC).

#### Trigonopeltastes geometricus

Taxon classificationAnimaliaColeopteraScarabaeidae

Schaum, 1841

Trigonopeltastes nigrinus Bates, 1889: 379. **Syn. n.**
Trigonopeltastes carus Bates, 1889, 381. **Syn n.**

##### Remarks.

Bates (1889) originally described *Trigonopeltastes nigrinus* as a variety of *Trigonopeltastes geometricus*, and Howden (1968) later gave *Trigonopeltastes nigrinus* full species status. Howden (1968) and Howden and Joly (1998) gave diagnostic characters for each species involving the size, indentation of pronotal triangle, elytral coloration, and pygidial markings but commented that these were based on the examination of “very few specimens” and that further study was needed. In examining longer series of *Trigonopeltastes geometricus*, I have observed that the diagnostic characters used by Howden (1968) to separate *Trigonopeltastes geometricus* and *Trigonopeltastes nigrinus* break down with a number of individuals exhibiting a blend of the supposed diagnostic characters used. Bates (1889) originally described *Trigonopeltastes carus* as a distinct species, which Howden (1968) synonymized with *Trigonopeltastes nigrinus*. Specimens that fall under any of the three preceding names all share the diagnostic characters used in the key and have significant lateral portions of the pygidium covered with yellow, cretaceous markings. Since I could find no justification for maintaining *Trigonopeltastes nigrinus* as a separate species, I am here synonymizing this name and *Trigonopeltastes carus* under *Trigonopeltastes geometricus*. Further study involving molecular data is desirable to test the hypothesis that *Trigonopeltastes geometricus* is a single, variable species that is distributed from Mexico to Bolivia.

##### Distribution.

*Trigonopeltastes geometricus* and its synonyms were previously recorded from Mexico, Belize, Guatemala, El Salvador, Nicaragua, Costa Rica, Panama, Venezuela, Colombia, Ecuador, and Bolivia (Howden 1968, Cave 1983, Maes et al. 1997, Howden and Joly 1998). The specimens detailed below represent a new country record for Honduras.

“HOND. Olancho / LaMuralla Pq Nac / 24-27 May 1995 / JE Wappes” (1 female - CMNC).

“HONDURAS: Yoro / PN Pico Pijol, 1300 / N15°09.4’ W87°37.6’ / 11.V.02, beating / H.Douglas” (1 female - CMNC).

“HONDURAS Atlantida / Dept., ex. log / Lancetilla Bot. Garden / 4 July 1995 / DC Carlson/FT Hovore” (1 female - DCCC).

#### Trigonopeltastes glabellus

Taxon classificationAnimaliaColeopteraScarabaeidae

Howden, 1988

##### Distribution.

This species was previously recorded from Mexico (Howden 1988). The specimens detailed below represent a new country record for Guatemala.

“Guat. Huehue Finca / Zapote, Rio Lagartero / 5-VI-1991 / Edmund F. Giesbert” (12 males – CMNC, EMEC).

#### Trigonopeltastes sallaei sallaei

Taxon classificationAnimaliaColeopteraScarabaeidae

Bates, 1889

##### Distribution.

This species was previously recorded from Mexico, El Salvador, Nicaragua, and Costa Rica (Howden 1968). The specimens detailed below represent new country records for Guatemala and Honduras.

“GUAT., Zacapa Sn / Lorenzo Rd 1500- / 1800'1-10 June / 1991 JE Wappes” (1 male - CMNC).

“GUAT.: BAJA VERAPAZ / 54.4km S. Purulha, / 850m, 1.VII.1993, / F. Génier, hand coll.” (2 males - CMNC).

“HONDURAS Olancho / Dept., P.N. La Muralla / ± 1200m, flowers / 1 July 1995 / DC Carlson/FT Hovore” (1 male - DCCC).

#### Trigonopeltastes simplex

Taxon classificationAnimaliaColeopteraScarabaeidae

Bates, 1889

##### Remarks.

The male of this species has never been formally described, and so I have included the description of key characters below with sexually dimorphic characters indicated.

##### Description of male

**Figures 52–55.**
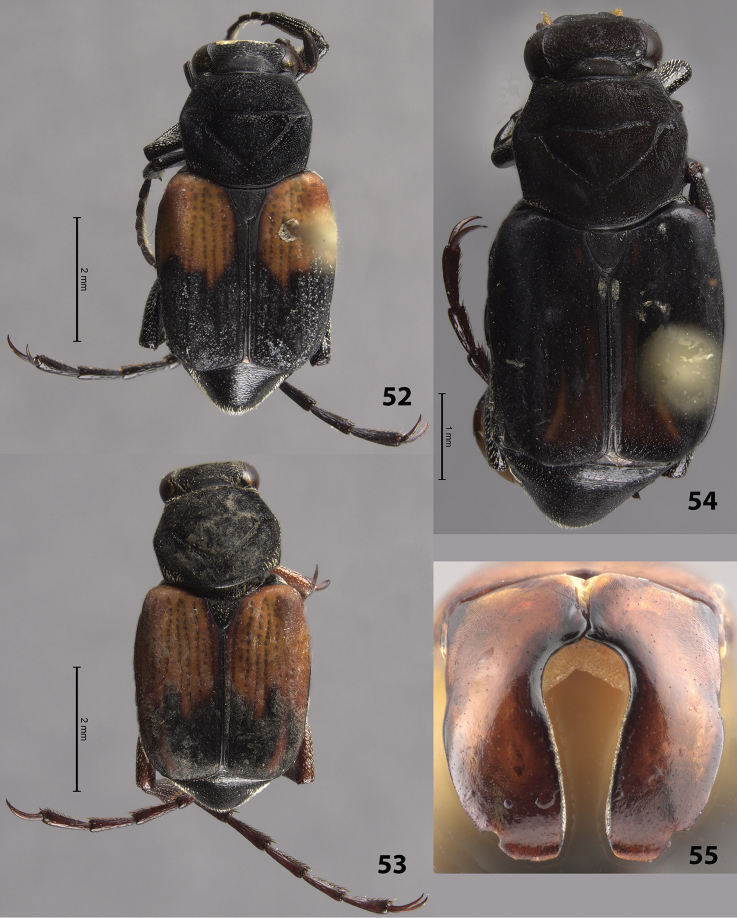
*Trigonopeltastes simplex* Bates, 1889, males from CMNC (label data: **52** “Guatemala, Zacapa / rd. to San Lorenzo, 4200’ / June 10-15, 1991 / E. Giesbert, coll.” **53** “GUAT. Baja Verapaz / 14.5km N.Salamá on / Pantín Rd. 1620 m / 23.V.1991 / H & A Howden” **54** “MEXICO, CHIAPAS / SUMIDERO CYN. 4000’ / JUNE 14 1987 / E. GIESBERT, COLL.”) **52–54** Dorsal habitus showing three variations of elytral color form **55** Parameres.

(Figs 52–55). Color: elytra and legs highly variable (see Figs 52–54); dorsal surface black with elytra all black, all dark orange, or with varying degrees of dark orange basally and black apically; each elytron lacking cretaceous markings or with short, transverse, cretaceous band on basal half of elytra suture and/or short cretaceous band adjacent to lateral edge approximately halfway between base and apex. Legs dark orange, black, or a combination of these colors. *Head*: Surface glabrous, without cretaceous markings. *Pronotum*: Surface of disc dull-black with shiny micropunctures (surface shiny in females). Marginal bead obsolete laterally, without cretaceous markings inside marginal bead (marginal bead complete and with inside cretaceous markings in females). Pronotal disc with more-or-less complete inverted triangle indented into surface and without cretaceous markings (female has cretaceous markings). *Elytra*: See above and Figs 52–54 for color variation. *Pygidium*: Surface without cretaceous makings (present laterally in females), disc with distinct ridges of concentric circles and some setae laterally and apically. *Venter*: Visible abdominal sternites 1–5 almost covered medially with cretaceous markings (except for small, central triangles); with short, white setae scattered throughout (or long setae just concentrated on sternite 5). *Legs*: Protibia with 2 teeth near apex (female with third medial tooth). *Parameres*: Robust with lateral notches towards apex (Fig. 55).

##### Distribution.

This species was previously recorded from Guatemala (Howden 1968). The specimens detailed below represent a new country record for Mexico. The Mexican specimens have some variation in the metathoracic leg characters and setal characters that bear further investigation.

“MEXICO, CHIAPAS / SUMIDERO CYN, 4000’ / JUNE 14 1987 / E. GIESBERT, COLL.” (1 male - CMNC).

“MEXICO. Chiapas. / Pq. Nac. Sumidero. / 1000m. 25.V.1990 / H.&A. Howden” (1 male - CMNC).

**Figure 56.**
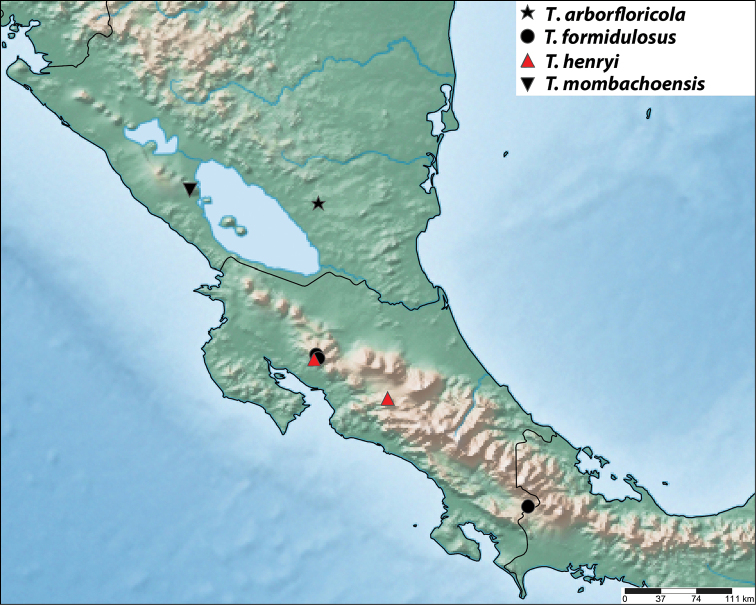
Distribution map in Nicaragua and Costa Rica for *Trigonopeltastes arborfloricola* sp. n. (★), *Trigonopeltastes formidulosus* sp. n. (●), *Trigonopeltastes henryi* sp. n. (▲), and *Trigonopeltastes mombachoensis* sp. n. (▼).

**Figure 57.**
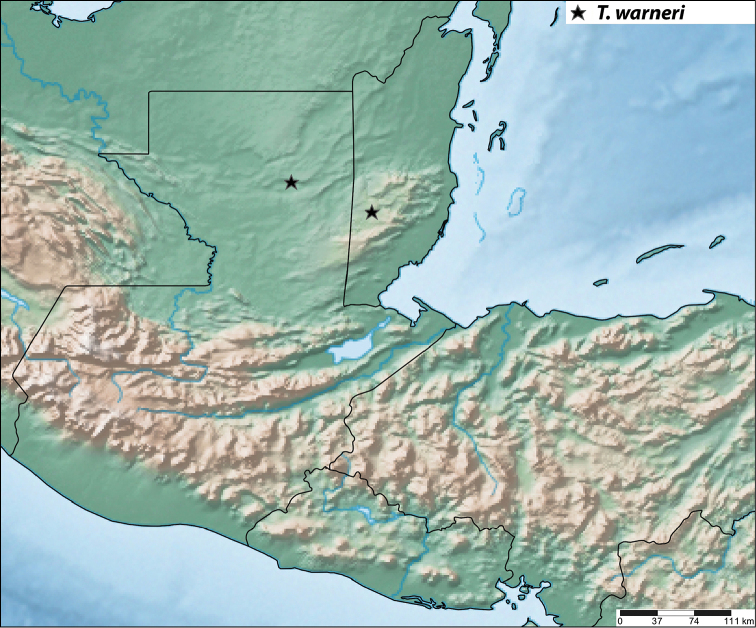
Distribution map in Guatemala and Belize for *Trigonopeltastes warneri* sp. n. (★).

#### Trigonopeltastes variabilis

Taxon classificationAnimaliaColeopteraScarabaeidae

Howden, 1968

##### Distribution.

This species was previously recorded from Mexico (Chiapas, San Luis Potosi, and Veracruz), Guatemala, and El Salvador (Howden 1968). The specimens detailed below represent a significant range extension north to the Mexican state of Tamaulipas and a new country record for Honduras.

“MEX Tamaulipas / Bocotoma Area 7 km / SSE Gomez Farias / June 1-4 1982 / J. E. Wappes” (1 male - CMNC).

“5 miles sse. of / Gomes Farias, / Tamaulipas, Mexico / July 19-20, 1970 / Murray, Phelps, / Hart, Schaffner” (1 male - CMNC).

“HONDURAS Olancho / Dept., P.N. La Muralla / ± 1200m, flowers / 1 July 1995 / DC Carlson/FT Hovore” (1 male - DCCC).

### Updated key to species of *Trigonopeltastes*

Modified from Howden (1968) and Howden and Joly (1998), and best used with the illustrations in those publications as a reference.

The male of *Trigonopeltastes femoratus* Howden is unknown and not included in the key.

The females of *Trigonopeltastes arborfloricola* sp. n., *Trigonopeltastes kerleyi* Ricchiardi, 2003, and *Trigonopeltastes thomasi* Howden & Ratcliffe, 1990 are unknown and not included in the key.

**Table:** 

1	Abdominal sternites 1–5 concave or flat; pygidium length approximately equal or greater than width. Males	**2**
–	Abdominal sternites 1–5 convex; pygidium width greater than length. Females	**27**
2	Head color mainly metallic green, pronotum green or blue matt (e.g., Fig. 22)	**3**
–	Head and pronotum not metallic, mainly black, tan, and/or brown (e.g., Figs 5, 11, 31, 41, 50)	**4**
3	Pronotum, scutellum, and elytra blue matt (Fig. 19)	***Trigonopeltastes henryi* Smith**
–	Pronotum and scutellum green matt, elytra tan	***Trigonopeltastes thomasi* Howden & Ratcliffe**
4	Pygidium without cretaceous markings, disc with scales and setae (e.g., Figs 6, 49)	**5**
–	Pygidium with cretaceous markings (at least along lateral edges), disc with scales and/or setae (e.g., Figs 12, 21, 32, 40)	**9**
5	Pronotal disc evenly covered with dense scales. Mexico	***Trigonopeltastes discrepans* Howden**
–	Pronotal disc with or without scales along margins and within impressed lines forming triangle, but scales not entirely covering pronotal surface	**6**
6	Pronotum with distinct, impressed, longitudinal midline (sometimes most easily seen running through triangle impression). Mexico to Guatemala	***Trigonopeltastes glabellus* Howden**
–	Pronotum without distinctly impressed longitudinal midline. Mexico to Nicaragua	**7**
7	Impressed margins of triangle on pronotum glabrous (Figs 52–54) (except small patch of setae sometime present at lateral corners). Mexico to Guatemala	***Trigonopeltastes simplex* Bates**
–	Impressed margins of triangle on pronotum with thick, scale-like setae (Figs 5, 50). Mexico and Nicaragua	**8**
8	Legs bicolored with orange basally and black apically (Fig. 47). Elytra completely orange. Mexico	***Trigonopeltastes aurovelutinus* Curoe**
–	Legs unicolored, black (Fig. 2). Elytra black with orange markings. Nicaragua	***Trigonopeltastes arborfloricola* Smith**
9	Clypeus with apical angles sharp, acute, distinctly reflexed. Durango, Mexico	***Trigonopeltastes truncatus* Howden**
–	Clypeus with apical angles not sharply angulate or distinctly reflexed	**10**
10	Elytral intervals 2–3 medially with transverse cretaceous markings forming (in conjunction with cretaceous marking of the elytral suture) an inverted “T” or “+” shape	**11**
–	Elytral intervals 2–3 without transverse cretaceous markings forming a medial “T” or “+” shape	**12**
11	Mesofemoral and metafemoral surfaces with uniform covering of short, scale-like setae. Mexico to Costa Rica	***Trigonopeltastes archimedes* Schaum**
–	Mesofemoral and metafemoral surfaces with thin, hair-like setae; without scales. Florida, United States of America	***Trigonopeltastes floridanus* (Casey)**
12	Clypeus largely reddish brown. Eastern United States of America	***Trigonopeltastes delta* (Forster)**
–	Clypeus largely black. Mexico to South America	**13**
13	Metatibia with inner surface distinctly modified with a basal swelling ending apically with a robust tooth. Mexico	***Trigonopeltastes deltoides* (Newman)**
–	Metatibia without modifications or teeth along the inner surface	**14**
14	Species occurring in South America	**15**
–	Species occurring in Mexico and Central America	**18**
15	Pronotal triangle lacking scales; penultimate abdominal sternites with conspicuous medial tuft of long, erect setae; metatibia on apical half of inner surface with dense, elongate brush of yellow setae (height of setae approximately equal to width of metatibia). Venezuela	***Trigonopeltastes barbatus* Howden & Joly**
–	Pronotal triangle with yellow scales (sometimes abraded); penultimate abdominal sternites without distinct medial tuft of setae; metatibia without dense elongate brush of setae on apical half (line of short setae sometime present but height of setae much less than width of metatibia)	**16**
16	Pygidium with cretaceous markings filling basolateral corners of disc. Venezuela, Colombia, Ecuador, Bolivia (also in Mexico and Central America)	***Trigonopeltastes geometricus* Schaum** (in part)
–	Pygidium with cretaceous markings not extending to basolateral corners of disc	**17**
17	Elytra with second and fourth intervals weakly elevated, shiny. Bahia, Brazil	***Trigonopeltastes kerleyi* Ricchiardi**
–	Elytra with second and fourth intervals flat, matt. Brazil, Paraguay, Argentina	***Trigonopeltastes triangulus* (Kirby)**
18	Pronotal triangle with apical, transverse line indicated only at midline, obsolete for approximately half of length; pygidial cretaceous markings interrupted basomedially. Mexico to El Salvador	***Trigonopeltastes variabilis* Howden**
–	Pronotal triangle with apical, transverse line complete or nearly complete; pygidial cretaceous markings usually not interrupted basomedially	**19**
19	Clypeus and vertex each with wide cretaceous markings on either side of midline; elytra without transverse cretaceous markings along lateral edge	***Trigonopeltastes wappesi* Howden**
–	Clypeus without cretaceous markings, vertex rarely with cretaceous markings; elytra usually with transverse cretaceous markings along lateral edge	**20**
20	Abdominal sternites 2–5 without long, erect setae standing out from appressed scales or fine recumbent setae on surface	**21**
–	Abdominal sternites 2–5 (sometimes only sternite 5) with long, erect setae standing out from appressed scales and recumbent setae on surface	**22**
21	Pygidium cretaceous except for thin, longitudinal strip along midline, apex slightly convex but not deflexed. Mexico to Panama (also in South America)	***Trigonopeltastes geometricus* Schaum** (in part)
–	Pygidium with small, lateral cretaceous spots (Fig. 12); apex with small area of tip deflexed on a roughly 90° angle compared to rest of pygidial surface. Costa Rica	***Trigonopeltastes formidulosus* Smith**
22	Abdominal sternite 5 only with long, erect setae; all other sternites without long, erect setae. Costa Rica and Panama	***Trigonopeltastes pontilis* Howden**
–	Abdominal sternites 2–5 all with long, erect setae	**23**
23	Clypeus basally with prominent patch of short, semierect, tan setae; vertex sometimes with cretaceous markings (Fig. 41); protibia with 2 prominent teeth along outer edge	**24**
–	Clypeus without prominent patch of setae (Fig. 31); protibia with 3 prominent teeth along outer edge	**25**
24	Protibia and mesotibia completely tan; elytra more tan than black. Mexico to Honduras	***Trigonopeltastes frontalis* Bates**
–	Protibia and mesotibia tan with prominent, black markings along external edge (Fig. 39); elytra more black than tan. Belize to Guatemala	***Trigonopeltastes warneri* Smith**
25	Metafemur with short, appressed scales or setae on much of exposed ventral surface (some long setae sometimes at basal posterior edge); pygidium with basal portion of cretaceous markings usually thick and rarely broken medially; elytra with transverse black mark across interval 2 and 3 adjacent to cretaceous marking on midline or intervals 2 and 3 completely orange adjacent to cretaceous marking on midline; elytra along lateral edges with 2 transverse cretaceous lines (basal line sometimes absent especially in individuals with mainly orange coloration on elytra). Mexico to Costa Rica	***Trigonopeltastes sallaei* Bates**
–	Metafemur with long, semierect setae (or very elongate flattened scales) on most of exposed ventral surface; pygidium with basal portion of cretaceous markings reduced and sometimes broken medially; elytra with interval 2 orange and interval 3 with black spot or line adjacent to cretaceous marking on midline; elytra along lateral edges with 1 transverse cretaceous line (sometimes absent) (Fig. 28)	**26**
26	Basal half of elytra with 1 transverse, solid orange line along base and medial orange spot near humerus (Fig. 28). Nicaragua	***Trigonopeltastes mombachoensis* Smith**
–	Basal half of elytra with 2 transverse, solid orange lines, one along base and one sub-basally. Mexico to Guatemala	***Trigonopeltastes intermedius* Bates**
27	Pygidium without cretaceous markings, disc with scales and setae	**28**
–	Pygidium with cretaceous markings (at least along lateral edges), disc with scales and/or setae (e.g., Figs 17, 27, 37, 44) (*Trigonopeltastes formidulosus* sometimes without cretaceous markings but pygidial disc appears glabrous)	**29**
28	Head, pronotum, and scutellum densely covered with scales. Mexico	***Trigonopeltastes discrepans* Howden**
–	Head, pronotum, and scutellum not densely covered with scales (mainly glabrous sometimes with patches of scales). Mexico to Guatemala	***Trigonopeltastes glabellus* Howden**
29	Species occurring in South America	**30**
–	Species occurring in the United States of America, Mexico, and Central America	**32**
30	Pygidium not evenly convex, medially often somewhat flattened transversely, apical third swollen. Brazil, Paraguay, Argentina	***Trigonopeltastes triangulus* (Kirby)**
–	Pygidium evenly convex	**31**
31	Pygidium with cretaceous markings across base continuous or narrowly divided; apex of pygidium lacking small, shiny, triangular tubercle. Venezuela, Colombia, Ecuador, Bolivia (also in Mexico and Central America)	***Trigonopeltastes geometricus* Schaum** (in part)
–	Pygidium with cretaceous markings distinctly separated basally; apex of pygidium with small, shiny, triangular tubercle. Venezuela	***Trigonopeltastes barbatus* Howden & Joly**
32	Pygidium bilobed either side of depressed midline, disc not evenly convex (Fig. 27). Costa Rica	***Trigonopeltastes henryi* Smith**
–	Pygidium not bilobed and without depressed midline, evenly convex (e.g., Figs 17, 37, 44)	**33**
33	Clypeus with apical angles sharp, acute, distinctly reflexed. Durango, Mexico	***Trigonopeltastes truncatus* Howden**
–	Clypeus with apical angles not sharply angulate or distinctly reflexed	**34**
34	Elytral intervals 2–3 medially with transverse, cretaceous markings forming (in conjunction with cretaceous marking of elytral suture) an inverted “T” or “+” shape	**35**
–	Elytral intervals 2–3 without transverse, cretaceous markings forming a medial “T” or “+” shape	**36**
35	Mesofemoral and metafemoral surfaces with some short, scale-like setae; elytron with prominent cretaceous markings along apex. Mexico to Costa Rica	***Trigonopeltastes archimedes* Schaum**
–	Mesofemoral and metafemoral surfaces with thin, hair-like setae; without scales; elytron without prominent cretaceous markings along apex. Florida, United States of America	***Trigonopeltastes floridanus* (Casey)**
36	Clypeus largely reddish brown. Eastern United States of America	***Trigonopeltastes delta* (Forster)**
–	Clypeus largely black. Mexico to Panama	**37**
37	Metafemur slender, no wider than apex of metatibia; pygidium with ovoid, basal, cretaceous markings distinctly separated from lateral margins. Costa Rica	***Trigonopeltastes femoratus* Howden**
–	Metafemur stocky, wider than apex of metatibia; pygidium with cretaceous markings extending to lateral margins	**38**
38	Length 10.5 mm or more. Mexico	***Trigonopeltastes deltoides* (Newman)**
–	Length 10.0 mm or less (with head in vertical position)	**39**
39	Pronotal triangle with apical, transverse line indicated only at midline, obsolete for approximately half of length; disc within triangle with fine to moderately-sized punctures. Mexico to El Salvador	***Trigonopeltastes variabilis* Howden**
–	Pronotal triangle with apical, transverse line complete or nearly complete; if incomplete then disc within triangle with large punctures	**40**
40	Clypeus and vertex each with wide, cretaceous markings either side of midline; elytra without transverse, cretaceous markings along lateral edge	***Trigonopeltastes wappesi* Howden**
–	Clypeus without cretaceous markings, vertex rarely with cretaceous markings; elytra usually with transverse cretaceous markings along lateral edge	**41**
41	Specimens with all of the following: pygidium with cretaceous markings well separated mediobasally; elytra with humeral area orange or reddish brown; pronotal surface shiny, not matt; clypeus with medial punctures or rugae not running longitudinally. Mexico to Guatemala	***Trigonopeltastes simplex* Bates**
–	Specimens without the above combination of characters	**42**
42	Clypeal length approximately equal to width, medial portion of disc with punctures or rugae running longitudinally (e.g., Fig. 16)	**43**
–	Clypeus wider than long, medial portion of disc with punctures or rugae running transversely or randomly (e.g., Figs 36, 45)	**45**
43	Pygidium with small, lateral cretaceous spots (Fig. 17) (sometimes absent)	***Trigonopeltastes formidulosus* Smith**
–	Pygidium with more extensive cretaceous markings covering either lateral and dorsal portions of pygidium or most of pygidium except midline (e.g., Figs 37, 44)	**44**
44	Pygidium with cretaceous markings rounded and even in thickness, cretaceous markings not reaching basolateral corners of pygidium. Costa Rica and Panama	***Trigonopeltastes pontilis* Howden**
–	Pygidium with cretaceous markings much thinner in some parts compared to others, cretaceous markings filling basolateral corners of pygidium. Mexico to Panama (also in South America)	***Trigonopeltastes geometricus* Schaum** (in part)
45	Femora tan to reddish brown	**46**
–	Femora black to dark brown (if tan, from Nicaragua)	**47**
46	Clypeus with a group of short, semierect setae on either side near lateral margins; elytral interval 2 with orange coloration extending apically past lateral cretaceous marking. Mexico to Honduras	***Trigonopeltastes frontalis* Bates**
–	Clypeus with inconspicuous, fine setae (Fig. 45); elytral interval 2 with orange coloration not extending apically beyond level of lateral cretaceous marking (Fig. 43). Belize to Guatemala	***Trigonopeltastes warneri* Smith**
47	Elytra with transverse, black mark across interval 2 and 3 adjacent to cretaceous marking on midline, or intervals 2 and 3 completely orange adjacent to cretaceous marking on midline; elytra along lateral edges with 2 transverse, cretaceous lines (basal line sometimes absent especially in individuals with mainly orange coloration on elytra); elytral humeral swelling usually either completely black or orange, without continuous orange line across base. Mexico to Costa Rica	***Trigonopeltastes sallaei* Bates**
–	Elytra with interval 2 orange and interval 3 with black spot or line adjacent to cretaceous marking on midline; elytra along lateral edges with 1 transverse, cretaceous line (sometimes absent); elytral humeral swelling with continuous orange line across the base (e.g., Figs 34–35)	**48**
48	Basal half of elytra with 1 lateral, solid orange line along base and medial orange spot sub-basally (Figs 34–35). Nicaragua	***Trigonopeltastes mombachoensis* Smith**
–	Basal half of elytra with 2 lateral, solid orange lines, one along base and one sub-basally. Mexico to Guatemala	***Trigonopeltastes intermedius* Bates**

### Checklist of the New World Trichiini

*Apeltastes* Howden, 1968

*Apeltastes chiapasensis* Howden, 1994 – Mexico

*Apeltastes elongatus* Howden, 1968 – Mexico

*Dialithus* Parry, 1849

*Dialithus magnificus* (Parry, 1849) – Mexico, Belize, Guatemala, Honduras, Nicaragua, Costa Rica

*Dialithus castaneipennis* Kraatz, 1897 (synonym)

*Dialithus scintillans* Howden, 1972 – Panama

*Giesbertiolus* Howden, 1988

*Giesbertiolus curoei* Ramírez-Ponce, 2014 – Panama

*Giesbertiolus festivus* (Howden, 1972) – Mexico

*Giesbertiolus linnaei* Krikken, 2008 – Costa Rica

*Giesbertiolus ornatus* Howden, 1988 – Costa Rica, Panama

*Gnorimella* Casey, 1915

*Gnorimella maculosa* (Knoch, 1801) – Canada, United States of America

*Trichius bigsbii* Kirby, 1827 (synonym)

*Gnorimus dissimilis* Gory & Percheron, 1833 (synonym)

*Iridisoma* Delgado-Castillo & Morón, 1991

*Iridisoma acahuizotlensis* Delgado-Castillo & Morón, 1991 – Mexico

*Paleotrichius* Poinar, 2011

*Paleotrichius dominicanus* Poinar, 2011 – Dominican Republic (fossil)

*Paragnorimus* Becker, 1910

*Peltotrichius* Howden, 1968 (synonym)

*Paragnorimus aenescens* (Bates, 1889) – Mexico

*Paragnorimus atratus* Smith, 2010 – Guatemala

*Paragnorimus glaseri* (Howden, 1971) – Guatemala

*Paragnorimus guatemalensis* Howden, 1970 – Guatemala

*Paragnorimus hondurensis* Smith, 2010 – Honduras, Nicaragua

*Paragnorimus howdeni* Smith, 2010 – Guatemala

*Paragnorimus linea* (Burmeister, 1841) – Mexico

*Trigonopeltastes quadrisignatus* Schaum, 1841 (synonym)

*Paragnorimus sambucus* Howden, 1970 – Mexico, Guatemala

*Paragnorimus velutinus* Becker, 1910 – Mexico

*Paragnorimus flohri* Becker, 1910 (synonym)

*Trichiotinus* Casey, 1915

*Trichinus* Kirby, 1827 (synonym)

*Trichiotinus affinis* (Gory & Percheron, 1833) – Canada, United States of America

*Trichius variabilis* Burmeister & Schaum, 1841 (synonym)

*Trichius mutabilis* Schaum, 1844 (synonym)

*Trichiotinus venticosus* Casey, 1915 (synonym)

*Trichiotinus parvulus* Casey, 1915 (synonym)

*Trichiotinus assimilis* (Kirby, 1837) – Canada, United States of America

*Trichius bistriga* Newman, 1838 (synonym)

*Trichius variabilis* Burmeister & Schaum, 1841 (synonym)

*Trichiotinus bibens* (Fabricius, 1775) – Canada, United States of America

*Trichiotinus lunulatus* (Fabricius, 1775) – United States of America

*Trichius viridulus* Fabricius, 1775 (synonym)

*Trichius virens* Gmelin, 1790 (synonym)

*Trichius mutabilis* Schaum, 1844 (synonym)

*Trichius semiviridis* Casey, 1914 (synonym)

*Trichius carolinensis* Casey, 1914 (synonym)

*Trichius rasilicaudus* Casey, 1915 (synonym)

*Trichius rufiventris* Casey, 1915 (synonym)

*Trichiotinus piger* (Fabricius, 1775) – Canada, United States of America

*Trichius drummond* Gory & Percheron, 1833 (synonym)

*Trichius rotundicollis* Kirby, 1837 (synonym)

*Trichiotinus reductus* Casey, 1915 (synonym)

*Trichiotinus rufobrunneus* (Casey, 1914) – United States of America

*Trichius obesulus* Casey, 1914 (synonym)

*Trichiotinus texanus* (Horn, 1876) – United States of America

*Trichiotinus monticola* Casey, 1915 (synonym)

*Trichiotinus intermedius* Casey, 1915 (synonym)

*Trichiotinus viridans* (Kirby, 1837) – Canada, United States of America

*Trichius variabilis* Burmeister & Schaum, 1841 (synonym)

*Trigonopeltastes* Burmeister & Schaum, 1840

*Archimedius* Kirby, 1827 (synonym)

*Euclidius* Kirby, 1827 (synonym)

*Roplisa* Casey, 1909 (synonym)

*Trigonopeltastes arborfloricola* Smith, 2016 – Nicaragua

*Trigonopeltastes archimedes* Schaum, 1841 – Mexico, Guatemala, El Salvador, Costa Rica

*Trigonopeltastes aurovelutinus* Curoe, 2011 – Mexico

*Trigonopeltastes barbatus* Howden & Joly, 1998 – Venezuela

*Trigonopeltastes delta* (Forster, 1771) – United States of America

*Trigonopeltastes deltoides* (Newman, 1838) – Mexico

*Trigonopeltastes discrepans* Howden, 1968 – Mexico

*Trigonopeltastes femoratus* Howden, 1968 – Costa Rica

*Trigonopeltastes floridanus* (Casey, 1909) – United States of America

*Trigonopeltastes formidulosus* Smith, 2016 – Costa Rica

*Trigonopeltastes frontalis* Bates, 1889 – Mexico, Belize, Guatemala, El Salvador, Honduras

*Trigonopeltastes geometricus* Schaum, 1841 – Mexico, Belize, Guatemala, El Salvador, Honduras, Nicaragua, Costa Rica, Panama, Venezuela, Colombia, Ecuador, Bolivia

*Trigonopeltastes nigrinus* Bates, 1889 (synonym)

*Trigonopeltastes carus* Bates, 1889 (synonym)

*Trigonopeltastes glabellus* Howden, 1988 – Mexico, Guatemala

*Trigonopeltastes henryi* Smith, 2016 – Costa Rica

*Trigonopeltastes intermedius* Bates, 1889 – Mexico, Guatemala

*Trigonopeltastes kerleyi* Ricchiardi, 2003 – Brazil

*Trigonopeltastes mombachoensis* Smith, 2016 – Nicaragua

*Trigonopeltastes pontilis* Howden, 1988 – Costa Rica, Panama

*Trigonopeltastes sallaei sallaei* Bates, 1889 – Mexico (eastern Mexico), Guatemala, El Salvador, Honduras, Nicaragua, Costa Rica

*Trigonopeltastes sallaei sinaloensis* Howden, 1968 – Mexico (northwestern Mexico from Sonora to Nayarit)

*Trigonopeltastes simplex* Bates, 1889 – Mexico, Guatemala

*Trigonopeltastes thomasi* Howden & Ratcliffe, 1990 – Mexico

*Trigonopeltastes triangulus* (Kirby, 1819) – Brazil, Paraguay, Argentina

*Trigonopeltastes nigra* Burmeister, 1846 (synonym)

*Trigonopeltastes truncatus* Howden, 1968 – Mexico

*Trigonopeltastes variabilis* Howden, 1968 – Mexico, Guatemala, El Salvador, Honduras

*Trigonopeltastes warneri* Smith, 2016 – Belize, Guatemala

*Trigonopeltastes wappesi* Howden, 1988 – Panama

## Supplementary Material

XML Treatment for Trigonopeltastes arborfloricola

XML Treatment for Trigonopeltastes formidulosus

XML Treatment for Trigonopeltastes henryi

XML Treatment for Trigonopeltastes mombachoensis

XML Treatment for Trigonopeltastes warneri

XML Treatment for Giesbertiolus ornatus

XML Treatment for Paragnorimus sambucus

XML Treatment for Trichiotinus bibens

XML Treatment for Trigonopeltastes archimedes

XML Treatment for Trigonopeltastes aurovelutinus

XML Treatment for Trigonopeltastes frontalis

XML Treatment for Trigonopeltastes geometricus

XML Treatment for Trigonopeltastes glabellus

XML Treatment for Trigonopeltastes sallaei sallaei

XML Treatment for Trigonopeltastes simplex

XML Treatment for Trigonopeltastes variabilis
